# Applications of nanomaterials for gastrointestinal tumors: A review

**DOI:** 10.3389/fmedt.2022.997123

**Published:** 2022-09-01

**Authors:** Rahul Kanaoujiya, Dipiti Porwal, Shekhar Srivastava

**Affiliations:** Synthetic Inorganic and Metallo-Organic Laboratory, Department of Chemistry, University of Allahabad, Prayagraj, India

**Keywords:** gastric cancer, gastrointestinal, nanotechnology, quantum dots, dendrimers, iron oxide nanoparticles, carbon nanotubes, nanoshells

## Abstract

Nanotechnology is the emerging and advance field of research for the diagnosis and treatment of various diseases. With the development of nanotechnology, different nanoparticles are used in the treatment of cancer due to their unique optical properties, excellent biocompatibility, surface effects, and small size effects. Nanoparticles are the particles which have the particular size from 1 to 100 nm. These nanoparticles are zero dimension, one dimension, two dimension and three dimension etc. In present scenario a variety of research is focused on the tailored synthesis of nanoparticles for medicinal applications that can be used for cancer treatment based on the morphology, composition, interaction with target cell. The gastrointestinal (GI) tumors are found one of the deadest cancer types with highest reoccurrence rates. The diagnosis and treatment of gastrointestinal cancer is very challenging due to its deep location and complicated surgery. Nanotechnology provides fast diagnosis and immediate treatment for the gastrointestinal disease. A variety of nanomaterials are used for the diagnosis and treatment of GI disease. Nanoparticles target directly to the tumor cell as diagnostic and therapeutic tools facilitating the identification and removal of tumor cells. A number of nanoparticles are developed for the uses are quantum dots (QDs), carbon nanotubes (CNTs), metallic nanoparticles (MNPs), Dendrimers etc. This review article gives an overview of the most promising nanomaterials used for the diagnosis and treatment of GI diseases. This review attempts to incorporate numerous uses for the most current nanomaterials, which have great potential for treating gastrointestinal diseases.

## Introduction

**Figure 1:**
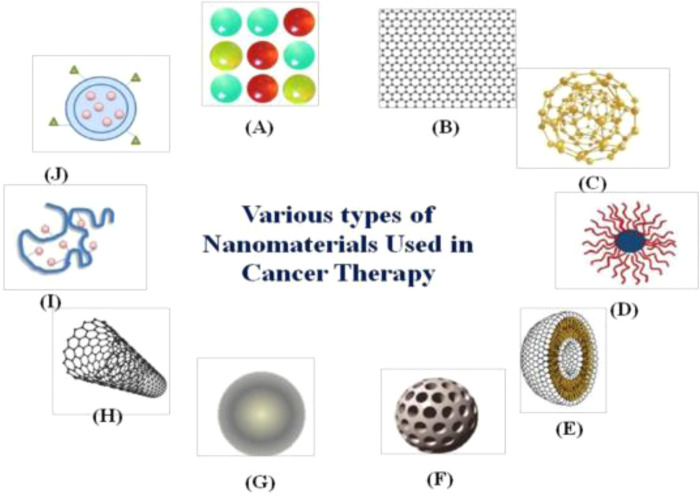
Diagrammatic representation of several kinds of nanomaterials used in cancer treatment. (**A**) Quantum dots, (**B**) graphene, (**C**) gold nanoparticles, (**D**) polymeric micelles, (**E**) liposomes, (**F**) silica nanoparticles, (**G**) magnetic nanoparticles, (**H**) carbon nanotubes, (**I**) polymerdrug conjugates, and (**J**) polymeric nanoparticles. Reproduced from (59). Copyright 2021, Medicina(MDPI).

An interdisciplinary research area that integrates chemistry, engineering, biology, and medicine is nanotechnology (1–3). There are several beneficial nanotechnological applications in cancer biology, including tools for the early identification of tumours and cancer biomarkers and the creation of treatment strategies that are not possible with conventional tools. The top ten new cancer cases in China in 2020 will be lung, colorectal, gastric, breast, liver, esophageal, thyroid, pancreatic, prostate, and cervical cancers, according to the “2020 Global Cancer Report” recently released by the World Health Organization's International Agency for Research on Cancer (IARC) (4). The majority of cases are gastrointestinal malignancies, which are strongly associated with people who consume a high sugar, low fibre diet, are sedentary, obese, drink alcohol, and smoke cigarettes (5, 6). Examining the treatment and diagnosis of gastrointestinal malignancies is crucial in order to lower the incidence and mortality of the disease as well as increase patient survival rates (7–9). The use of nanotechnology in cancer research has given the scientific community optimism for the creation of revolutionary cancer therapy techniques (10). Significant efforts have been made to develop novel diagnostic and therapeutic approaches for enhancing patient quality of life and lengthening survival because gastrointestinal cancers account for more than 55% of cancer-related fatalities (11, 12). Numerous disciplines including chemistry, engineering, biology, and medicine are paying attention to nanotechnology. Several types of nanomaterials used in cancer treatment (13, 14) such as shown in Figure 1. (A) quantum dots, (B) graphene, (C) gold nanoparticles, (D) polymeric micelles, (E) liposomes, (F) silica nanoparticles, (G) magnetic nanoparticles, (H) carbon nanotubes, (I) polymerdrug conjugates, and (J) polymeric nanoparticles.

The gastrointestinal cancer is one of the leading causes of cancer death worldwide. In 2017 more than 864 to 989 patients died from this cancer and numbers are increasing each year. It arises due to the difficulty in diagnosis and treatment of tumors within GI tract. The GI cancers have the worst type of all the cancers and have lowest rate of survival. It is important to develop high accuracy in early diagnostic and effective treatment for it. Currently nanotechnology is receiving attention in versatile fields of engineering science and medicine. Due to high aspect ratio and smaller size the properties of nanomaterials are more prominating than the bulk material. Nanoparticles have significant biocompatibility and offer potential applications in therapeuticand diagnostic purposes. Nanoparticles are used as imaging agent, drug delivery agents.

The high biocompatibility and programmability of nanoparticle (NP)-based technologies present prospects in therapeutic and diagnostic applications, particularly in cancer. NPs are effectively used in a variety of applications, including imaging agents, photothermal therapy, recognition, and medication and gene delivery. NPs have benefited from their high histocompatibility and adaptable properties. The GI tract is a desirable target for nanotechnological applications because it can modify the features of NPs through changes in pH, pressure, and bacterial content (15, 16). The upper and bottom portions of the GI tract make create a muscular tube that is about 9 m long. The mouth, pharynx, oesophagus, stomach, and first portion of the small intestine make up the upper GI tract, while the remaining portions of the small intestine and the large intestine are found in the lower GI tract.Food digestion, nutrition absorption, and waste product excretion are the GI tract's three primary tasks. A sizable portion of cancer cases worldwide are related to the gastrointestinal tract. Together, the various kinds of gastrointestinal cancer account for 40% of all malignancies that are diagnosed globally (18, 19). The use of nanoparticles in the treatment of cancer is reviewed in this review as they become a cutting-edge modality. The objective is for scientists to be able to expand on what has already been developed while addressing problems and flaws that have been found.

### Overview of recent developments in gastrointestinal therapy

**Figure 2:**
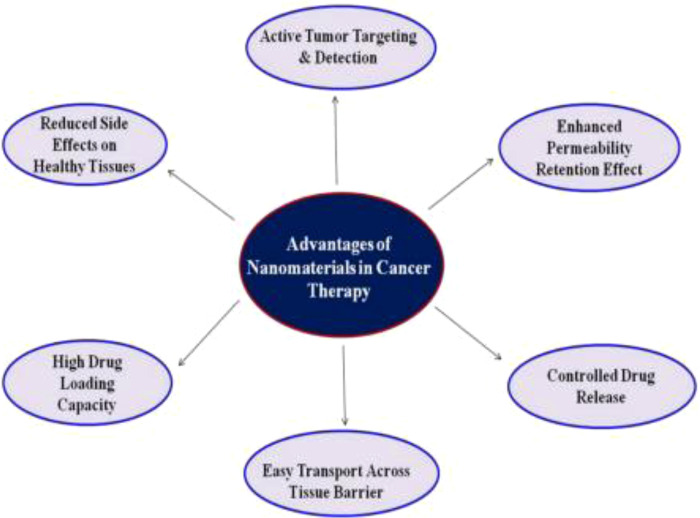
Systematic representation of Nanomaterials’ advantages in cancer treatment. Reproduced from (59). Copyright 2021, Medicina (MDPI).

The second, third and fourth most frequent types of cancer in terms of cancer mortality as well as the third, fourth, and fifth most frequent types of cancer in terms of incidence are all considered to be part of the large category of cancer subtypes known as gastrointestinal malignancies (20, 21). Gastrointestinal malignancies account for roughly 30% of all cancers worldwide and account for about 35% of cancer-related deaths. The form of therapeutic delivery and the potential level of toxicities these may produce are two of the biggest challenges in the treatment of cancer (22, 23). Since patients must stay in the hospital for extended periods of time during treatment, intravenous chemotherapy is not only uncomfortable for the patient but also expensive. Nanomaterials' distinctive characteristics allow them to function normally in the challenging GI environment. Drug interactions with the GI tract can be impacted by the size, shape, and surface functionalization of the NPs (24). Another crucial component is the non-specific interactions between the biomolecules and the nanodevices. To minimise nonspecific interactions and eliminate the influence of mucosal or GI cells, NPs can be coated with hydrophilic polymers. Various nanodevices that are being researched for gastrointestinal cancer are shown in Table 1. Advancement of nanomaterial in cancer treatment such as controlled drug release, high drug loading capacity, easy transport across tissue barrier, enhanced permeability retention effect, active tumor targeting & detection (25, 26) and reduced side effects on healthy tissues are shown in Figure 2.

**Table 1:** Diagrammatic representation of nanoparticles presently being investigated for gastrointestinal cancer.

GI Cancer	NP Type	Application	References
Gastric	Nanoshell	Targeted Drug Delivery	(38)
Liver	Carbon Nanotubes	Tumor Localization	(39)
Colorectal,Gastric	Polymers	Controlled Drugs delivery systems	(40)
Colorectal	Dendrimers	Targeted Drug Delivery	(41)
Gastric,liver,Colorectal	Gold Nanoparticles	Targeted Drug Delivery	(42)
Colorectal,Gastric,Liver	SPION	companion diagnostics	(43)

### Quantum dots (QDs)

QDs are semiconductor nanoparticles having an inorganic element at their core and a metal shell surrounding it. They are a type of nanocrystal. QDs have sizes between 2 and 10 nm (27, 28). QDs have attractive properties such multispectral tunability, great sensitivity, and no need for lasers. They also have steady fluorescence with straightforward excitation. Whole-blood assays are possible thanks to QDs' red/infrared hues. If injection poses a hazardous risk, there could be another issue with employing QDs *in vivo*.

Although changes have been made to lessen potential toxicity, more research is needed to identify the proper clinical applicability. Due to their special qualities, such as improved permeability and retention effect and nanoscale vehicle features with high imaging agent capacity, QDs are thought to be the best technique for cancer targeting and imaging (29, 30). In the field of immune his to chemistry, which is frequently employed in the diagnosis of malignant tumours, the usage of multicolor QD probes is being studied more and more. As a trustworthy indicator of disease progression and therapeutic response, QDs-based multiplexed biomarker detection has received a lot of interest. By analysing the main elements of the tumour stroma, including tumour infiltrating macrophages, QDs conjugated with various biomarkers may also be used for simultaneous prognosticator detection to predict medical outcomes in gastric cancer (31, 32).

### Dendrimers

Dendrimers are artificial complex nanostructures that have branched, concentric layers encircling an inner core. A dendrimer can have its size, branching length, shape, and surface capabilities altered to serve a variety of purposes. Dendrimers have sizes that range from 1 to 10 nm (44, 45). A variety of proteins that are now recognised by individual ELISA tests are now being detected using dendrimers, which is proving to be very effective at doing so. Additionally, dendrimer nanoparticles have been developed, which can be used with a single probe for imaging with both MRI and NIR fluorescence modalities. The development of dendrimers as non-viral delivery vectors took use of their superior and distinctive trait of increased permeability. Dendrimers are a promising carrier for the delivery of targeted antitumor genes for cancer therapy since they can successfully transfer the tumour necrosis factor gene into colorectal adenocarcinoma cells to prevent the growth of colorectal cancer without obvious damage on animals (46, 47). A member of the non-receptor tyrosine kinase protein family called cSrc is overexpressed and activated in a variety of human cancer cells and EGFR-dependent downstream genes. PAMAM dendrimers complexed with cSrc antisense significantly lowered cSrc (48).

### Gastric cancer

For the treatment and diagnostics of stomach cancer, nanomedicine has demonstrated its usefulness and potential. Due to the high prevalence of gastric cancer not only in the US but also globally, this is of utmost importance. According to research, 738,000 people die from gastric cancer each year, making up 8% of all new cancer cases (49, 50). Numerous research on gastric cancer are being conducted, despite the fact that many therapeutic nanocarriers have not yet received approval for usage in clinical. The use of fluorescent magnetic nanoparticles is a striking illustration of how useful nanoparticles can be in treating stomach cancer. The limitations of imaging and chemical-based diagnostic methods have been addressed by substantial research on nanoparticles.

Gold nanostructures are an effective structure for cancer photothermal therapy because they are easy to make. However, compared to photothermal therapy alone, a combination of several modalities has been shown to be more effective in treating cancer (51). A number of modalities have been demonstrated to be more potent at treating cancer than photothermal therapy alone, though. Another strategy for treating gallbladder cancer that has recently been established involves the use of nanoparticles coupled to chemotherapy and localised to the tumour location by the use of magnetic fields (52). A magnetic thread was inserted into the tumour tissue, which allowed the nanoparticles to be specifically targeted to the tumour site. It was discovered that dose-responsive tumour size reductions happened (53).

Currently, GNPs are crucial in the management of gastrointestinal malignancies. In contrast to previous cancer treatments, photothermal therapy can target tumour tissue to heat it while sparing healthy tissues (54, 55). The plasmonic nanoparticles are supplied to the tumour cells or tissue after being exposed to NIR radiation, where the absorbed light is transformed into heat and irreparably damages the neighbouring diseased tissues. Many different nanoparticles are employed in PTT but GNPs in particular can passively accumulate in tumour tissue (56). GNPs' critical roles in the diagnosis of gastrointestinal cancers are shown in Table 2.

**Table 2:** Table shown GNPs’ critical roles in the diagnosis of gastrointestinal cancers.

Detection	Nanoparticle Size	Particle name	Main results	References
Gastric cancer	71.40 nm	Fe3O4@Au@b-CD	targeting the cells of gastric cancer cells	(33)
Gastric cancer	∼58 nm	AuNCs@SiO2-FA	Targeting gastric cancer	(34)
Hepatoma carcinoma	95.4 ± 2.4 nm	Ac-PE-AuNPs	More accumulated in the healthy liver than in the area of necrosis	(35)
Colorectal cancer	54 ± 11 nm	cmHsp70-AuNPs	Targeting to colorectal cancer	(36)
Pancreatic cancer	∼50 nm	GoldMag	MRI is feasible to quantify delivery	(37)

### Iron oxide nanoparticles

Due to the extreme conditions any drug delivery vehicle must endure before releasing its pharmacological payload, medication delivery to the gastrointestinal (GI) tract is very difficult. Knowing where the capsule is exactly and when to administer an external stimulation is crucial since efficient targeted drug delivery systems frequently rely on external stimuli to cause release. We offer a medication delivery method for the GI tract based on coating typical gelatin capsules with a model eicosane-superparamagnetic iron oxide nanoparticle composite coating (60). This coating is activated utilising magnetic hyperthermia as an on-demand release mechanism to heat and melt the coating. Iron oxide nanoparticles (IONPs) have several uses in biomedicine, particularly in bioimaging.

### Carbon nanotubes

Single-walled carbon nanotubes (SWCNTs) have a wide range of industrial and commercial applications, which has significantly increased their production. SWCNTs are probably entering these systems through manufacturing waste streams and leaching since aquatic habitats frequently serve as sinks for both point and non-point source pollutants. Characterizing potential effects of SWCNTs on aquatic habitats is therefore becoming more and more important. CNTs are 2D nanoscale material with high strength, electrical conductivity. These properties make them applicable for diagnosis of cancer cells and also are drug delivery agent. Further functionlised single walled and multiwalled CNTs are used as high resolution contrast agent (61). SWCNTs have smaller size and smaller band gap so they are more suitable for diagnosis and treatment purpose. SWCNTs functionilised with Gadolinium have high resolution and penetration. SWCNTs are generally not overtly hazardous to fish, according to studies that have looked into this issue. Oral exposures by gavage and eating haven't elicited much of a reaction, despite some evidence suggesting that waterborne exposures to these compounds may cause respiratory stress. However, the challenge of detecting and quantifying these substances *in vivo* has been one of the largest obstacles to thorough toxicity assessment, including uptake, distribution, and sublethal toxicity of SWCNTs.

### Nanoshells

Nanoshells are the composite of metal shell and a nanoconductive cores. They change the Plasmon resonance by changing the size of metal shell and nonmetal core. Nanoshells enhance the potential of traditional contrast agent for the imaging of tumors with higher sensitivity and resolution. Nanoshells are essential for spectroscopic and cancer research (62). The one-step and two-step techniques used in the manufacture of nanoshells are both quite straightforward. Silica nanoshells are the most common type employed in the field of molecular encapsulation, whereas metal nanoshells are used in cancer treatment. Different ligands including organic compounds, polymers, and surfactants, stabilise nanoparticles. Core-shell particles are nanoparticles that have a shell that protects their surface in addition to their core and that has unique features of its own. According to its intended application, this shell may be composed of metals or oxides. Along with stabilising colloidal dispersions, this kind of coating also enables customization and alteration of the particle's physical characteristics, including its optical, magnetic, and catalytic properties. Extreme conditions show that oxide-protected nanoparticles are more stable (63).

## Conclusion

As evidenced by a wealth of scientific literature, cancer is a quickly developing illness that poses a challenge to existing targeted medication treatments. This implies that even if a technique has been created to reliably produce these nanosized particles to give a therapeutic treatment, the cancer may possibly display resistance in the future. To design diagnostic and therapeutic instruments and devices, nanostructures and nanotechnology-based gadgets are actively being developed. Nanoparticles can be modified to display unique properties at the cellular, atomic, and molecular levels. Their size ranges from 1 to 100 nm. Continuous changes have been brought about by quick breakthroughs and advancements. An overview of the existing nanotechnologies for the detection and management of gastrointestinal cancers is given in this article. We provide a summary of the use of nanotechnologies in GI cancer diagnosis and treatment now. The high specificity and sensitivity of nanodevices makes them biocompatible and harmless. There is still study to be done as well as opportunities for future research. Nanomaterials have a very promising future and appeal to every branch of contemporary research. The main categories of NPs that could be used in gastroenterology have been outlined in this review. A fast growing field of study is the use of nanotechnology in medicine. It looks very possible that nanotechnology will soon play a significant part in diagnosing and treating gastroenterological problems.

## Data Availability

The original contributions presented in the study are included in the article, further inquiries can be directed to the corresponding author.
